# A Review of the Occurrence and Recovery of Rare Earth Elements from Electronic Waste

**DOI:** 10.3390/molecules29194624

**Published:** 2024-09-29

**Authors:** Binjun Liang, Jihan Gu, Xiangrong Zeng, Weiquan Yuan, Mingjun Rao, Bin Xiao, Haixiang Hu

**Affiliations:** 1 Ganzhou Key Laboratory of Mine Geological Disaster Prevention and Control and Ecological Restoration, School of Resources and Civil Engineering, Gannan University of Science and Technology, Ganzhou 341000, China; liangbinjun1205@163.com (B.L.); gujihan@gnust.edu.cn (J.G.); zengxr986@163.com (X.Z.); ywqsdut@163.com (W.Y.); 2 Chongyi Green Metallurgy New Energy Co., Ltd., Ganzhou 341300, China; 3 School of Minerals Processing & Bioengineering, Central South University, Changsha 410083, China; mj.rao@csu.edu.cn

**Keywords:** electronic waste, rare earth elements, recovery, green technologies, hydrometallurgy

## Abstract

Electronic waste (e-waste) contains valuable rare earth elements (REEs) essential for various high-tech applications, making their recovery crucial for sustainable resource management. This review provides an overview of the occurrence of REEs in e-waste and discusses both conventional and emerging green technologies for their recovery. Conventional methods include physical separation, hydrometallurgy, and pyrometallurgy, while innovative approaches such as bioleaching, supercritical fluid extraction, ionic liquid extraction, and lanmodulin-derived peptides offer improved environmental sustainability and efficiency. The article presents case studies on the extraction of REEs from waste permanent magnets and fluorescent powders, highlighting the specific processes involved. Future research should focus on developing eco-friendly leaching agents, separation materials, and process optimization to enhance the overall sustainability and efficiency of REE recovery from e-waste, addressing both resource recovery and environmental concerns effectively.

## 1. Introduction

**Figure 1:**
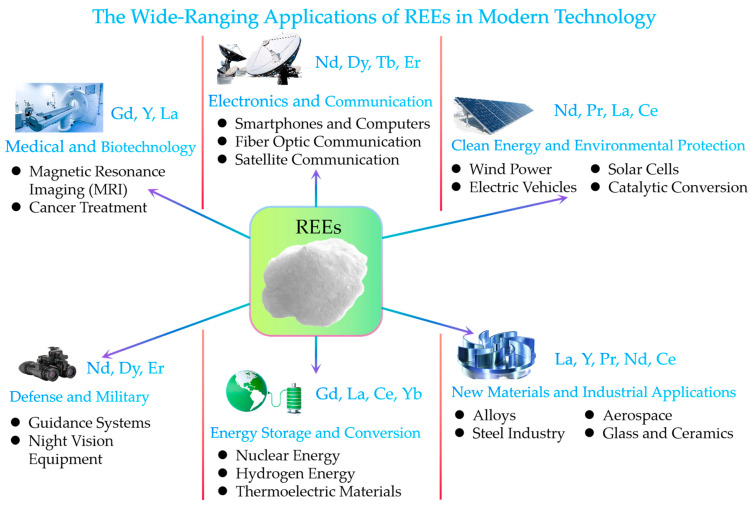
The indispensable role of REEs in modern technology.

Rare earth elements (REEs), comprising 17 metallic elements including the lanthanides, scandium, and yttrium, have gained significant attention due to their unique electronic, magnetic, and optical properties. These properties make REEs indispensable in a wide range of modern technologies and applications, as shown in Figure 1. REEs are extensively used in clean energy (wind turbines, electric vehicle motors, and energy-efficient lighting systems) [1], consumer electronics (screens, speakers, and other components) [2], transportation (catalytic converters, fuel cells, and lightweight alloys) [3], defense (precision-guided munitions, night vision devices, and communication systems) [2,3], and advanced materials (ceramics, glasses, and superconductors) [4,5,6]. The unique magnetic properties of REEs, particularly neodymium and dysprosium, are essential for producing high-performance permanent magnets used in wind turbine generators and electric vehicle motors [1,2]. The growing demand for REEs across these diverse technological fields has led to increased interest in developing efficient and sustainable methods for their extraction and separation [7,8,9].

The rapid advancement and widespread adoption of consumer electronics have led to a significant increase in electronic waste (e-waste) generation globally [10]. It is estimated that the world generates over 50 million tons of e-waste annually, with an expected growth to 74.7 million tons by 2030 and potentially reaching higher figures by 2050 [11]. E-waste, which includes discarded smartphones, computers, televisions, and other electronic devices, contains a variety of valuable materials, including REEs [12,13]. Studies have shown that the concentration of REEs in e-waste is often higher than that found in natural ores. For example, hard disk drives contain up to 2500 ppm of neodymium and 500 ppm of dysprosium, while fluorescent lamps contain up to 20,000 ppm of yttrium and 1000 ppm of europium [14,15]. This revised description ensures accuracy and aligns with the latest available data on e-waste generation and the presence of REEs in electronic waste.

The potential value of REE recovery from e-waste is twofold. First, it provides an alternative source of REEs, reducing the dependence on primary mining and the associated environmental impacts [16,17]. Second, recovering REEs from e-waste contributes to the circular economy by minimizing waste disposal, conserving natural resources, and promoting sustainable development [16,18]. However, the complex composition of e-waste and the lack of efficient recycling technologies pose challenges to the widespread adoption of REE recovery from this secondary resource [19].

This review aims to provide a comprehensive overview of the occurrence and recovery of REEs from e-waste. The article is structured as follows: Section 2 discusses the occurrence characteristics of REEs in various types of e-waste and the factors influencing their distribution; Section 3 reviews the current technologies and approaches for REE recovery from e-waste, including physical separation, hydrometallurgical extraction, pyrometallurgical extraction, and emerging green technologies; Section 4 presents case studies of REE recovery from typical e-waste, such as waste permanent magnets and fluorescent powders; Section 5 highlights the existing problems and future perspectives in this field, emphasizing the urgent need for establishing recovery systems and regulations, optimizing recovery technologies and synergizing resource utilization with environmental protection; and Section 6 concludes a review, with key findings and outlooks. To maintain brevity and clarity, several abbreviations are used throughout this article to refer to key terms and techniques related to the recovery of REEs from e-waste. Readers can find the full forms of these abbreviations in the table provided at the end of the article.

## 2. Occurrence Characteristics of Rare Earth Elements in Electronic Waste

E-waste contains a wide variety of materials, including valuable metals such as gold, silver, copper, and REEs [19]. The occurrence and distribution of REEs in e-waste depend on the type of electronic device and its components [20]. Understanding the distribution and concentration of REEs in different types of e-waste is crucial for developing effective recovery and recycling strategies [21]. This section discusses the common types of e-waste and their REE content, as well as the factors influencing the occurrence of REEs in e-waste.

### 2.1. Common Types of Electronic Waste and Their Rare Earth Content

E-waste encompasses a diverse range of discarded electronic devices, including computers, smartphones, televisions, and home appliances. Table 1 presents the typical REE content in various types of e-waste.

As shown in Table 1, hard disk drives and fluorescent lamps contain the highest concentrations of REEs, particularly neodymium, dysprosium, yttrium, and europium. The permanent magnets in hard disk drives, such as NdFeB (neodymium–iron–boron) magnets, are the primary source of neodymium and dysprosium [13,22]. The phosphors in fluorescent lamps, on the other hand, are rich in yttrium and europium [22,23]. Smartphones, computers, and televisions also contain various REEs, although at lower concentrations compared to hard disk drives and fluorescent lamps. The REEs in these devices are mainly found in components such as screens, speakers, and vibration motors [23,24].

### 2.2. Factors Influencing the Occurrence of Rare Earth Elements in Electronic Waste

**Figure 2:**
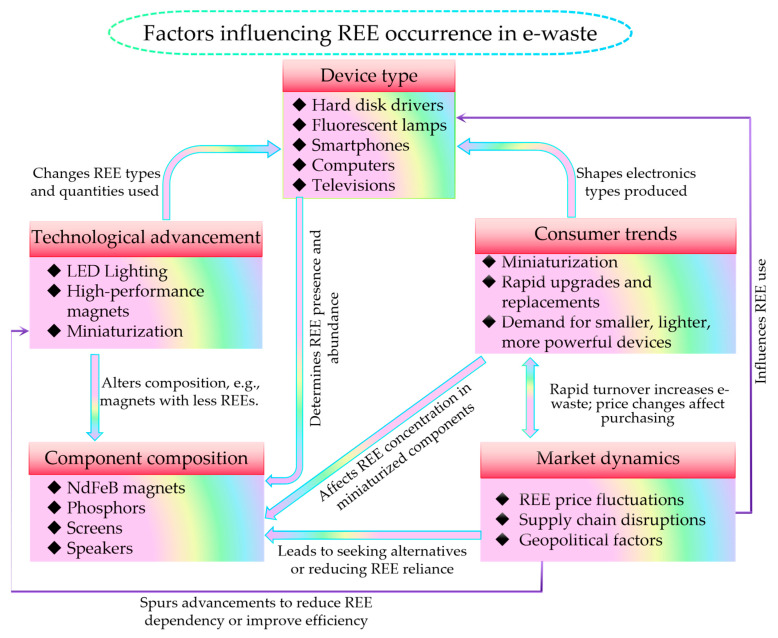
An overview of the sources, impacts, and recovery options for rare earth elements in electronic waste.

The occurrence and concentration of REEs in e-waste are influenced by various factors, which can be broadly categorized into five main aspects: device type, component composition, technological advancements, consumer trends, and market dynamics. Understanding these factors is crucial for developing effective strategies to manage and recover REEs from e-waste. Figure 2 provides an overview of the sources, impacts, and recovery options for REEs in e-waste, highlighting the importance of considering these factors in the context of e-waste management.

In the following subsections, we will discuss each of these factors in detail, exploring how they influence the presence and distribution of REEs in various types of electronic waste, along with relevant data and examples to support the analysis.

#### 2.2.1. Device Type

As introduced in Section 2.1, the type of electronic device plays a significant role in determining the concentration and distribution of REEs in e-waste. Different devices, such as hard disk drives (HDDs), fluorescent lamps, smartphones, computers, and televisions, contain varying amounts of REEs due to the specific components they use.

For example, HDDs contain NdFeB magnets, which are a primary source of neodymium and dysprosium. A study highlighted that HDDs are significant contributors to REE content in e-waste due to these magnets [25]. Fluorescent lamps use phosphors that are rich in yttrium and europium, but the transition to LED lighting has reduced the demand for these REEs in lighting applications [26,27]. Smartphones and computers contain various REEs in their screens, speakers, and other components, with smartphones often containing dysprosium, terbium, and neodymium in their small, high-performance magnets [28]. Televisions utilize REEs in their display technologies, such as phosphors in cathode ray tubes and LEDs in modern screens [29].

#### 2.2.2. Component Composition

The specific composition of components within electronic devices determines the type and quantity of REEs present. For instance, NdFeB magnets found in HDDs and other electronic devices are significant sources of neodymium and dysprosium [30]. Phosphors used in lighting and display technologies contain yttrium, europium, and terbium, with the specific composition of these phosphors dictating the presence of REEs in the e-waste [31]. Dielectric ceramics used in capacitors and other electronic components often incorporate REEs like lanthanum and cerium [32].

#### 2.2.3. Technological Advancements

Technological progress can significantly influence the use of REEs in electronic devices. As new technologies emerge, the types and quantities of REEs required may change, or alternative materials may replace REEs altogether. For example, the transition from fluorescent lamps to LED lighting has reduced the demand for REEs used in fluorescent lamps, such as yttrium and europium [33].

Advancements in magnet technology, such as the development of high-performance magnets with optimized microstructures and compositions, may lead to a reduction in REE content without compromising performance. Research into rare earth-free permanent magnets is ongoing to further decrease reliance on REEs. For instance, sintered ferrite and Mn–Al–C magnets are being developed as alternatives to RE-based magnets, offering significant environmental and economic benefits [34]. Efforts to find alternatives to REEs in various applications are driven by the need to mitigate supply risks and reduce costs [35].

#### 2.2.4. Consumer Trends

Consumer behavior and preferences play a significant role in the occurrence of REEs in e-waste. The increasing demand for compact, high-performance electronic devices has led to the miniaturization of components, resulting in a higher concentration of REEs in these devices. Moreover, the rapid turnover of electronic devices, particularly in the consumer electronics market, has contributed to the growing volume of e-waste containing REEs [36,37].

Consumers’ awareness and attitudes towards e-waste recycling are crucial factors in the effective management of REEs in e-waste. Research has shown that individual attitudes, social norms, and perceived behavioral control significantly influence consumers’ intentions to recycle e-waste [38]. Furthermore, personality traits and social influences can impact consumers’ willingness to engage in e-waste recycling programs [37,38].

To tackle the e-waste challenge and enhance REE recovery, various strategies have been suggested, such as the following:

- Implementing user-friendly, household-scale e-waste recycling and REE recovery systems could encourage e-waste mining among all stakeholders, including industry, households, and regulatory bodies [11]. These systems provide convenient recycling options, which can increase participation rates and reduce the amount of e-waste sent to landfills.
- Promoting eco-friendly products and educating consumers about the significance of proper e-waste disposal through green marketing initiatives can shape consumer behavior and foster sustainable practices [37,38]. By raising awareness about the environmental consequences of e-waste and the importance of recycling, companies can encourage more responsible consumption habits.
- Continuous research into rare earth-free alternatives and the optimization of existing technologies can potentially reduce the dependence on REEs in electronic devices, thus alleviating the e-waste problem in the long run [11,36]. The development of new technologies may enable the production of electronic devices that are more easily recyclable and contain fewer REEs, reducing the environmental impact of e-waste.
- Introducing policies that promote sustainable consumption patterns, extended producer responsibility, and efficient e-waste collection and recycling systems can help address the challenges associated with REEs in e-waste [37,39]. Government regulations can mandate manufacturers to design products with recyclability in mind, establish e-waste collection and recycling infrastructure, and create incentives for consumers to participate in e-waste recycling programs.

Addressing consumer trends and implementing these strategies requires a collaborative approach involving all stakeholders, including consumers, manufacturers, and policymakers [37,40]. By working together to tackle the complex challenges associated with REEs in e-waste, we can move towards a more sustainable and circular economy, mitigating the environmental impact of e-waste and improving the recovery and recycling of valuable REEs from discarded electronic devices.

#### 2.2.5. Market Dynamics

**Figure 3:**
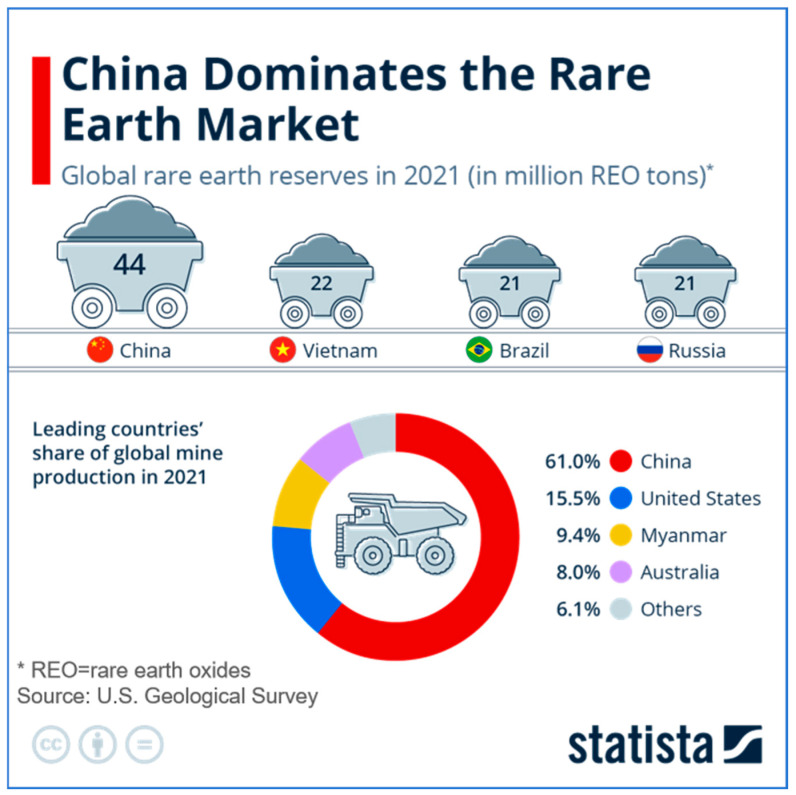
Global rare earth reserves and leading countries’ share of mine production in 2021 (from Statista) [41].

The global market for REEs and their applications in the electronics industry significantly influences the occurrence of these elements in e-waste. As shown in Figure 3, China dominates the global rare earth market, with reserves of 44 million REO tons in 2021, followed by Vietnam, Brazil, and Russia [41]. This dominant position allows China to exert significant control over the global REE supply chain, affecting the availability and use of REEs in electronic devices. In 2021, China accounted for 61.0% of the global rare earth mine production, followed by the United States (15.5%), Myanmar (9.4%), and Australia (8.0%) [41].

Geopolitical issues, such as export restrictions by China, can disrupt the global REE supply chain, impacting the production of electronic devices and the generation of e-waste. The growing demand for electric vehicles, wind turbines, and other green technologies further increases the need for REEs, putting additional pressure on the supply chain [42].

To address the challenges associated with REE supply and demand, several strategies have been proposed, such as exploring new REE deposits, developing efficient REE recycling techniques, improving substitution technologies, and reducing the number of critical REEs used in devices [43,44]. However, implementing these measures faces significant challenges due to environmental factors and an unbalanced market, requiring efforts from both governments and REE companies [43].

Fluctuations in REE prices can significantly impact the profitability of companies in the electronics and clean energy sectors. For instance, a Japan–Bangladesh joint venture medical device manufacturing company reported its first annual net profit after introducing a ‘Benefit-Sharing Model’ in its supply chain operations, which helped address issues related to price fluctuations and limited supplier sources [45].

The market dynamics of REEs also influence the occurrence of these elements in e-waste. During periods of high REE prices or supply shortages, manufacturers may seek to reduce their reliance on these elements or find alternative materials, potentially affecting the composition of electronic devices and the resulting e-waste [46].

By considering the influence of market dynamics alongside device type, component composition, technological advancements, and consumer trends, stakeholders can better predict and address the challenges associated with the occurrence of REEs in e-waste. This comprehensive approach will contribute to the sustainable recovery and recycling of these critical elements, supporting a more circular economy and ensuring a stable supply of REEs for future technologies.

## 3. Recovery and Extraction Techniques for Rare Earth Elements from E-Waste

As introduced in Section 1, the recovery of REEs from e-waste is crucial for sustainable resource management and reducing dependence on primary mining. Various techniques have been developed to extract and recover REEs from e-waste, each with its own advantages and challenges. Figure 3 presents a comprehensive schematic of the key methods covered in this review, categorized by their primary extraction principles and environmental impact.

**Figure 4:**
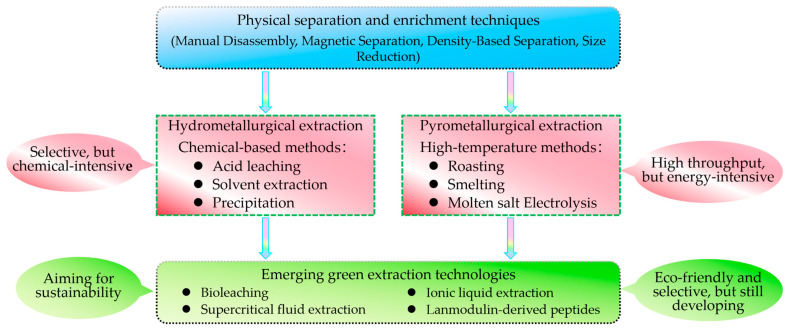
Overview of REE recovery methods from e-waste: physical separation, hydrometallurgical extraction, pyrometallurgical extraction, and emerging green technologies.

As illustrated in Figure 4, the methods discussed in this review span a wide range of extraction principles, from mechanical separation to chemical and biological processes. The environmental impact of these methods varies, with some traditional techniques like hydrometallurgy and pyrometallurgy having higher environmental footprints due to their use of hazardous chemicals and high energy consumption. In contrast, emerging green technologies such as bioleaching, supercritical fluid extraction, ionic liquid extraction, and lanmodulin-derived peptides offer more environmentally friendly alternatives by reducing the use of harmful chemicals and minimizing energy consumption.

In the following subsections, we will delve into the details of each method, discussing their principles, advantages, challenges, and recent advancements, starting with physical separation and enrichment techniques in Section 3.1.

### 3.1. Physical Separation and Enrichment Techniques

Physical separation and enrichment techniques form the crucial first step in recovering REEs from e-waste. These methods concentrate REE-containing components and separate them from other materials without altering their chemical composition, reducing the volume of material requiring more intensive chemical processing. To better understand the various physical separation techniques used in REE recovery from e-waste, let us examine their key characteristics, advantages, and applications in Table 2.

As shown in Table 2, each technique has its specific principles, advantages, and applications in the REE recovery process. The process typically begins with manual disassembly, followed by magnetic separation to exploit the magnetic properties of REE-containing components. Density-based separation methods are then utilized, and finally, size reduction techniques such as crushing and grinding are applied, followed by sieving to classify materials by size for subsequent processing [51,52].

The importance of these techniques is underscored by the staggering volume of global e-waste generation, estimated at 20–50 million metric tonnes annually, containing a wealth of technologically relevant REEs [47]. Recent beneficiation studies on bottom ash and fly ash samples have demonstrated the effectiveness of density separation, magnetic separation, and froth flotation in concentrating REEs, findings that are particularly relevant to e-waste processing [49,50].

The patent landscape for REE recovery from end-of-life electronic equipment reveals a diverse array of methods, including hydrometallurgy, pyrometallurgy, and biometallurgy or bioleaching [53,54]. This broad spectrum of patented techniques indicates significant interest and investment in developing efficient recovery processes, with physical separation methods playing a foundational role [47].

By employing these physical separation and enrichment techniques, the e-waste recycling industry can effectively concentrate REEs and minimize the volume of material requiring more intensive chemical processing. This not only improves the efficiency of subsequent extraction steps but also contributes to the overall sustainability and economic viability of REE recovery from e-waste.

Physical separation and enrichment techniques play a vital role in the initial stages of REE recovery from e-waste by concentrating REE-containing components and reducing the volume of material for subsequent chemical processing [51]. However, these methods face challenges such as high labor intensity and limited applicability to certain e-waste types [55]. Future research should prioritize the development of automated sorting systems, enhance the selectivity of physical separation methods, and explore innovative ways to integrate these techniques with advanced chemical extraction processes, fostering a more efficient and sustainable REE recovery ecosystem [56,57].

### 3.2. Hydrometallurgical Extraction Techniques

Following the physical separation and enrichment of REE-containing materials from e-waste, hydrometallurgical techniques are commonly employed to extract and purify the desired elements. Hydrometallurgy involves the use of aqueous solutions to selectively dissolve and recover target metals from solid materials. The key steps in hydrometallurgical REE extraction include leaching, solvent extraction, and precipitation or crystallization.

Leaching is the process of dissolving REEs from the enriched e-waste materials using acidic or alkaline solutions. Common leaching agents include sulfuric acid (H_2_SO_4_), hydrochloric acid (HCl), and nitric acid (HNO_3_) [58,59]. The choice of leaching agent depends on the specific REE-containing material and the presence of other elements that may interfere with the extraction process. Factors such as the leaching time, temperature, and solid-to-liquid ratio also play a crucial role in optimizing REE recovery [58].

After leaching, solvent extraction is often used to selectively separate and purify the dissolved REEs from the leach solution. This process involves the use of organic extractants that selectively bind to REE ions, allowing their separation from other dissolved metals [60]. Common extractants used in REE solvent extraction include di-(2-ethylhexyl) phosphoric acid (D2EHPA), 2-ethylhexyl phosphonic acid mono-2-ethylhexyl ester (PC-88A), and tributyl phosphate (TBP) [59].

Finally, the purified REE solutions are subjected to precipitation or crystallization to obtain solid REE products. This can be achieved by adjusting the pH of the solution or by adding precipitating agents such as oxalic acid or ammonium bicarbonate [61,62]. The precipitated REE compounds are then filtered, washed, and calcined to obtain high-purity REE oxides. Table 3 provides an overview of these key hydrometallurgical techniques used in REE extraction from e-waste, including precipitation along with leaching and solvent extraction.

The selection of appropriate hydrometallurgical techniques depends on the specific e-waste material, the targeted REEs, and the desired purity of the final products [63]. Recent research has expanded beyond traditional hydrometallurgical processes, focusing on eco-sustainable and innovative techniques to improve REE recovery rates, minimize environmental impact, and reduce overall costs. These advancements include the use of algal sorbents for sustainable REE recovery from e-waste [13], the application of various liquid membrane technologies for efficient and environmentally friendly REE extraction [64], and the exploration of ionic liquids for REE recovery from coal combustion products [65]. Additionally, there is a growing emphasis on incorporating REE recovery into broader circular economy frameworks [66] and developing methods for extracting these elements from low-grade and secondary resources [67]. These diverse approaches reflect a shift towards more sustainable, efficient, and environmentally conscious REE recovery methods.

In summary, hydrometallurgical techniques play a vital role in the extraction and purification of REEs from e-waste following physical separation and enrichment. By leveraging the principles of leaching, solvent extraction, and precipitation, and by adopting innovative and eco-friendly approaches, the e-waste recycling industry can efficiently recover these critical elements, contributing to a more sustainable and circular economy. As the demand for REEs continues to grow, the development of advanced hydrometallurgical processes will be crucial in ensuring a stable and environmentally responsible supply of these essential materials.

### 3.3. Pyrometallurgical Extraction Techniques

Pyrometallurgical techniques, which involve the use of high-temperature processes to extract and purify REEs from e-waste, are often used in combination with hydrometallurgical techniques to achieve optimal recovery rates and product purity. The main pyrometallurgical processes used in REE recovery from e-waste include roasting, smelting, and molten salt electrolysis.

Roasting is a high-temperature process that converts REE-containing compounds into oxides, making them more amenable to subsequent leaching or separation processes [68]. This technique is particularly useful for treating e-waste materials containing REE phosphors or oxides [69,70]. The roasting process can be carried out in the presence of additives, such as sodium carbonate (Na_2_CO_3_) or sodium hydroxide (NaOH), to enhance the conversion efficiency and selectivity [68,71].

Following roasting, smelting involves melting the e-waste material at high temperatures to concentrate the REEs in a molten phase, which can then be separated from the slag. This process is often used to recover REEs from metallic e-waste components, such as NdFeB magnets [72]. The smelting process can be optimized by adjusting parameters such as temperature, flux composition, and reducing agents to maximize REE recovery and minimize energy consumption [73,74,75].

Another important pyrometallurgical technique is molten salt electrolysis, an electrochemical method that uses high-temperature molten salts as the electrolyte to extract REEs from e-waste [72,76]. This method is particularly suitable for recovering REEs from fluorescent lamp phosphors, as it can efficiently separate REEs from the calcium and phosphate matrix [57]. The process involves dissolving the e-waste material in a molten salt bath, typically consisting of lithium fluoride (LiF) and REE fluorides, followed by an electrolytic deposition of the REEs at the cathode [72,77].

Table 4 provides an overview of the key pyrometallurgical techniques used in REE recovery from e-waste, along with their principles, advantages, and applications. Despite the benefits of these techniques, pyrometallurgical processes face challenges related to high energy consumption and potential environmental impacts [78,79]. To address these issues, ongoing research focuses on developing more energy-efficient and environmentally friendly pyrometallurgical processes, such as the use of microwave heating [78] and the optimization of flux compositions to minimize waste generation [80].

In conclusion, pyrometallurgical techniques play a complementary role to hydrometallurgical methods in the recovery of REEs from e-waste. By leveraging high-temperature processes such as roasting, smelting, and molten salt electrolysis, the e-waste recycling industry can effectively extract REEs from a wide range of materials, including phosphors, oxides, and metallic components. As the demand for sustainable REE sources grows, the development of advanced and eco-friendly pyrometallurgical processes will be essential to maximize recovery rates and minimize environmental impacts, ultimately contributing to a more circular economy.

### 3.4. Emerging Green Extraction Technologies

As the demand for REEs continues to grow and the environmental impacts of traditional extraction methods come under increasing scrutiny, there is a pressing need for the development of green and sustainable extraction technologies. These emerging approaches aim to minimize the use of hazardous chemicals, reduce energy consumption, and improve the overall eco-efficiency of REE recovery from e-waste [82,83]. This section highlights some of the most promising green extraction technologies currently being explored by researchers and industry stakeholders.

#### 3.4.1. Bioleaching

Bioleaching is a promising alternative to conventional hydrometallurgical processes, employing microorganisms to extract REEs from e-waste materials [84]. This approach leverages the ability of certain bacteria and fungi to solubilize metals through the production of organic acids and other metabolites [85]. Bioleaching offers several advantages over traditional methods, including lower energy consumption, reduced chemical usage, and the potential for selective metal recovery [22,86]. Key factors influencing the bioleaching process include temperature, particle size, pH value, medium composition, and microbial species [85].

**Figure 5:**
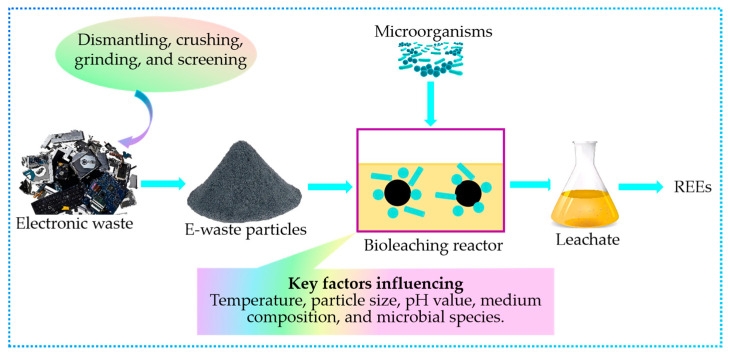
Schematic illustration of the bioleaching process for REE recovery from e-waste.

Figure 5 illustrates the key steps involved in the bioleaching process for REE recovery from e-waste. The process begins with the pretreatment of e-waste, which typically involves dismantling, crushing, grinding, and screening to obtain e-waste particles of a suitable size [84]. These particles are then introduced into a bioleaching reactor, where they interact with microorganisms [22]. The microorganisms produce organic acids and other metabolites that dissolve the REEs present in the e-waste particles [84,85], resulting in a leachate enriched with REEs [22,86].

Recent studies have demonstrated the effectiveness of bioleaching in extracting REEs from various e-waste streams. For example, researchers have successfully recovered REEs from fluorescent lamp phosphors using levulinic acid, with leaching efficiencies exceeding 98.7% under optimal conditions [87]. Gluconobacter oxydans has shown promising results in extracting REEs from spent fluid catalytic cracking (FCC) catalysts, with leaching efficiencies up to 56% in batch processes and 51% in continuous bioreactor systems [22].

To further enhance the sustainability and cost-effectiveness of bioleaching, researchers are exploring the use of agricultural wastes as substrates for fermentation. Studies have shown that biolixiviants generated from potato wastes and corn stover can be effective in REE recovery, potentially reducing costs compared to glucose-based processes. A techno-economic analysis indicated that a corn stover-based bioleaching plant would have 20% lower total costs than a potato wastewater-based plant, and a potato wastewater-based plant should have 18% lower total costs compared to a glucose-based plan [86].

The optimization of bioleaching parameters is crucial for achieving high REE recovery rates and selectivity. Ongoing research focuses on enhancing process efficiency by fine-tuning factors such as temperature, pH, medium composition, and duration [85,88]. For instance, a study on red mud leaching found that optimal conditions of a liquid-to-solid ratio of 40, temperature of 70 °C, and duration of 60 h led to 100% leaching of REEs excluding Sc [87].

Despite the promising potential of bioleaching as a green extraction technology for REEs, challenges remain. These include improving leaching efficiencies for various REE-bearing materials and developing microbial strains tailored to specific substrates [85,89]. Future research efforts should focus on addressing these challenges and scaling up bioleaching processes for industrial applications [90]. The development of continuous culture systems for acid-producing organisms like G. oxydans could provide organic acids in quantities and at rates useful for commercial bioleaching [86]. Additionally, exploring the potential of various microorganisms for REE bioleaching from different solid matrices could open up new avenues for efficient REE recovery and recycling [91]. By overcoming these obstacles, bioleaching can help reduce the supply risk and market dependency for REEs while contributing to more sustainable and environmentally friendly economic practices [85,89].

Bioleaching, as an emerging green technology, offers a promising alternative to conventional hydrometallurgical processes for REE recovery from e-waste. By harnessing the power of microorganisms, bioleaching can potentially reduce energy consumption, minimize the use of hazardous chemicals, and enable the selective recovery of target metals. However, the efficiency of bioleaching processes depends on various factors, such as the type of e-waste, microbial strains, and process parameters. Future research should focus on optimizing bioleaching conditions, developing microbial consortia tailored to specific e-waste streams, and investigating the potential synergies between bioleaching and other sustainable technologies, such as biosorption or bioreduction, to further enhance the efficiency and selectivity of REE recovery from e-waste.

#### 3.4.2. Supercritical Fluid Extraction

Supercritical fluid extraction (SFE) is an innovative technology that utilizes fluids above their critical points to extract REEs from e-waste. The most commonly used fluid is supercritical carbon dioxide (scCO_2_), which offers several benefits, including low toxicity, non-flammability, and easy separation from extracted components [92].

SFE has been successfully applied to the recovery of REEs from fluorescent lamp phosphors [93], NdFeB magnets [72], and NiMH batteries [94]. The high selectivity and efficiency of SFE, combined with its reduced environmental footprint, make it a promising green extraction technology for REE recovery from e-waste [93,94].

**Figure 6:**
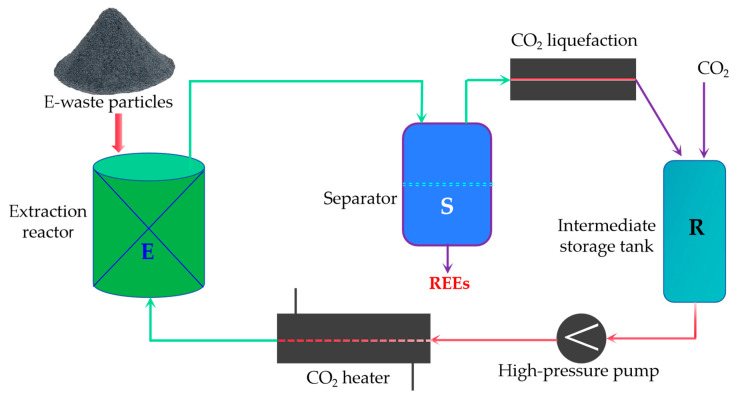
Schematic diagram of supercritical fluid extraction of rare earth elements from e-waste.

Figure 6 illustrates the process of REE recovery from e-waste using supercritical fluid extraction. The pretreated e-waste particles are introduced into the extraction reactor (E). CO_2_ is converted into supercritical CO_2_ (scCO_2_) by pressurization using a high-pressure pump and heating using a CO_2_ heater. Inside the extraction reactor, the scCO_2_ interacts with the e-waste particles, selectively dissolving and extracting the REEs. The REE-laden scCO_2_ is then transported to the separator (S), where a decrease in pressure causes the REEs to precipitate. The gaseous CO_2_ is recovered, liquefied, and recycled back to the system. The extracted REEs are collected for further processing.

The optimization of process parameters, such as pressure, temperature, and extraction time, is crucial for achieving high REE recovery rates and purity [94]. For instance, a study on the extraction of REEs from NdFeB magnets found that increasing the pressure from 15 MPa to 25 MPa at a constant temperature of 60 °C enhanced the extraction efficiency from 70% to 95% [72].

Another advantage of SFE is its ability to tune the solvent properties of scCO_2_ by introducing co-solvents or chelating agents. This approach can further enhance the selectivity and efficiency of REE extraction. For example, the addition of tri-n-butyl phosphate (TBP) as a co-solvent improved the extraction of REEs from fluorescent lamp phosphors, achieving recovery rates of up to 98% [93].

Despite the promising potential of SFE for REE recovery from e-waste, challenges remain in terms of process optimization and scaling up for industrial applications. Future research should focus on investigating the extraction mechanisms, developing novel co-solvents and chelating agents, and designing continuous-flow SFE systems for large-scale REE recovery from various e-waste streams.

#### 3.4.3. Ionic Liquid Extraction

Ionic liquids (ILs) are a class of low-melting-point salts that have gained attention as environmentally friendly alternatives to conventional organic solvents in REE extraction processes. ILs possess unique properties, such as low volatility, high thermal stability, and tunable selectivity, making them well suited for the selective extraction of REEs from e-waste leachates [95].

**Figure 7:**
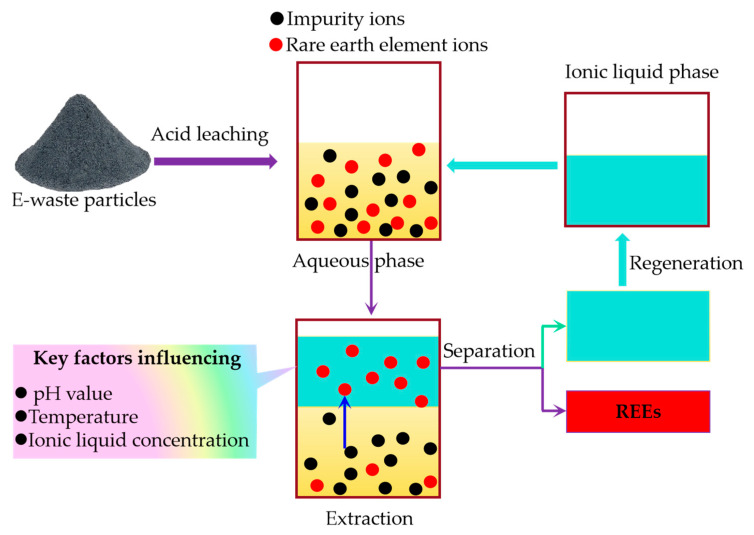
Schematic diagram of ionic liquid extraction of rare earth elements from e-waste.

Figure 7 illustrates the process of REE recovery from e-waste using ionic liquid extraction. The process begins with the acid leaching of e-waste particles, which dissolves the REEs and other impurities into the aqueous phase. The resulting leachate, containing a mixture of rare earth element ions and impurity ions, is then brought into contact with the ionic liquid phase. The resulting leachate, containing a mixture of rare earth element ions and impurity ions, is then brought into contact with the ionic liquid phase. During the extraction process, the REE ions are selectively transferred from the aqueous phase to the ionic liquid phase, while the majority of the impurity ions remain in the aqueous phase. This selective extraction is driven by the unique properties of ionic liquids, such as their tunable selectivity towards REE ions [96]. Key factors influencing the extraction efficiency and selectivity include pH value, temperature, and ionic liquid concentration [97].

After extraction, the REE-enriched ionic liquid phase undergoes a separation process to recover the concentrated REEs. The regenerated ionic liquid is then recycled back to the extraction stage, minimizing waste generation and enhancing the environmental sustainability of the process [96].

Recent studies have explored the use of various ILs for REE recovery from NdFeB magnets and fluorescent lamp phosphors. For example, Dupont et al. [98] demonstrated the successful extraction of neodymium and dysprosium from NdFeB magnet leachates using the ionic liquid betainium bis(trifluoromethylsulfonyl)imide ([Hbet][Tf2N]). This IL has shown promise due to its ability to selectively extract REEs through a proton-exchange mechanism, making it effective even under milder conditions compared to traditional high-temperature processes.

Similarly, high extraction efficiencies for europium and yttrium from fluorescent lamp phosphor leachates have been reported using various ionic liquids. For instance, a study demonstrated the separation of yttrium and europium from fluorescent lamp waste powder using undiluted thiocyanate ionic liquids, specifically trihexyl(tetradecyl)phosphonium thiocyanate ([C101][SCN]) and tricaprylmethylammonium thiocyanate ([A336][SCN]), with [C101][SCN] exhibiting the best extraction performance through a counter-current extraction process [99]. Another study utilized a supported ionic liquid membrane containing a mixture of imidazolium ionic liquid, tributyl phosphate (TBP), and D2EHPA for the selective extraction of yttrium ions from fluorescent lamp waste, optimizing conditions to achieve high extraction efficiencies [100].

In summary, ionic liquid extraction is a promising green technology for the recovery of REEs from e-waste. With their unique properties and tunable selectivity, ionic liquids offer a more environmentally friendly and efficient alternative to conventional solvent extraction processes. As research continues to advance in this field, ionic liquid extraction is poised to play a significant role in the sustainable recovery of critical REEs from e-waste.

#### 3.4.4. Lanmodulin-Derived Peptides

Lanmodulin-derived peptides are an emerging biotechnology that has gained considerable attention due to their high selectivity for REEs, regeneration potential, and sustainability. This approach mimics the function of the lanmodulin protein using simpler and more easily manipulated and optimized short peptides [101]. Among these peptides, LanM1, derived from the EF-hand loop I of lanmodulin, has shown particularly promising results in terms of its strong REE binding affinity both in solution and when immobilized on solid matrices [102,103].

**Figure 8:**
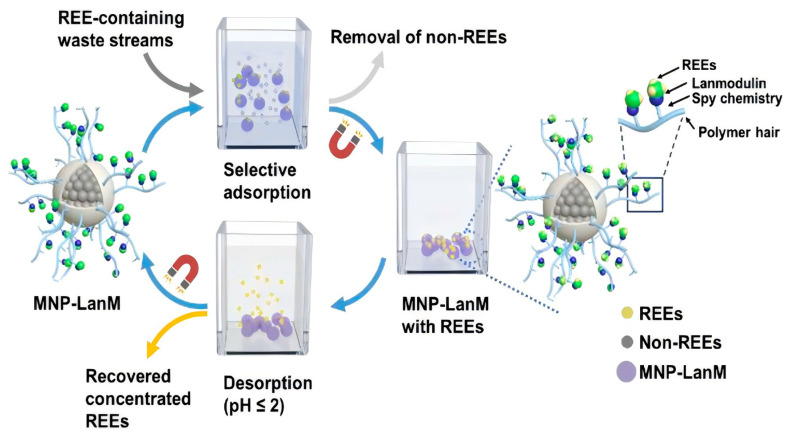
Schematic of the MNP-LanM biosorption process for selective REE recovery. Reprinted with permission from Ye et al. [102]. Copyright 2023 Royal Society of Chemistry.

A recent study by Ye et al. [102] demonstrated the application of a novel MNP-LanM biosorbent for the selective separation and recovery of REEs from waste streams. This biosorbent immobilizes Lanmodulin-SpyCatcher (LanM-Spycatcher) on the surface of SpyTag-functionalized magnetic nanoparticles (MNPs). A schematic of the MNP-LanM biosorption process for selective REE recovery is illustrated in Figure 8. The MNP-LanM biosorbent utilizes the strong binding affinity of lanmodulin-derived peptides to selectively adsorb and concentrate REEs from complex waste streams, such as coal fly ash leachate and geothermal brine. The biosorbent exhibited remarkable performance characteristics, including high adsorption activity, fast adsorption kinetics, efficient desorption, excellent selectivity for REEs in the presence of various non-REEs, enhanced stability, reusability, and efficient magnetic separation. The effectiveness of this biosorbent was demonstrated by a remarkable 967-fold increase in REE purity, highlighting its potential as a sustainable and efficient method for REE extraction from waste streams.

The development of lanmodulin-derived peptides for REE recovery continues to advance, with researchers exploring various immobilization techniques and optimizing peptide sequences. Verma et al. [103] investigated the performance of LanM1 for binding REEs both in solution and when surface-bound. The study employed multiple analytical techniques, including circular dichroism (CD), nuclear magnetic resonance (NMR), molecular dynamics (MD) simulations, isothermal titration calorimetry (ITC), and quartz crystal microbalance with dissipation (QCM-D), to characterize LanM1’s binding affinity towards various REEs such as Ce(III), Nd(III), Eu(III), and Y(III). The research demonstrated that LanM1 exhibits stronger REE binding affinity compared to its scrambled version in both solution and surface-immobilized states, even under low-pH conditions. These findings underscore the potential of lanmodulin-derived peptides for advanced separation technologies in REE recovery from electronic waste and other sources.

These insights contribute to the ongoing development of bioinspired strategies for REE recovery, highlighting the importance of peptide design and immobilization techniques in optimizing selectivity and efficiency. The focus on surface-bound peptides is particularly relevant for the development of practical REE separation and recovery systems from waste streams.

As research continues to advance in the field of lanmodulin-derived peptides, it is evident that these biomolecules have the potential to revolutionize REE recovery from e-waste and other low-grade sources. The high selectivity, regeneration potential, and compatibility with various immobilization techniques make lanmodulin-derived peptides a promising green technology for the development of advanced separation systems. Future research efforts should focus on the further optimization of peptide sequences, an exploration of novel immobilization methods, and scaling up these technologies for industrial applications. With continued progress in this field, lanmodulin-derived peptides are poised to play a crucial role in addressing the growing need for sustainable and efficient REE recovery from electronic waste and beyond [103].

### 3.5. Comparison of REE Recovery Methods

To facilitate the selection and optimization of suitable REE extraction methods for specific applications, it is essential to compare the various techniques in terms of their efficiency, purity, and limitations. Table 5 provides a detailed comparison of these recovery methods, considering key aspects such as recovery percentage, recovered form, environmental impact, energy use, cost-effectiveness, and pros/cons.

The various REE extraction methods exhibit significant differences in terms of efficiency, purity, and limitations. Traditional hydrometallurgy can achieve high recovery rates and purity but faces issues such as high chemical consumption and the generation of acidic wastewater [108]. Bioleaching is relatively eco-friendly but suffers from slow processes and limited product purity [109]. While supercritical fluid extraction and ionic liquid extraction can yield high-purity REEs, they are constrained by high-pressure requirements and the cost of ionic liquids, respectively [110]. Pyrometallurgy offers high recovery rates but is energy-intensive and poses risks of harmful emissions [111]. The efficiency and applicability of physical separation techniques vary depending on the type of waste.

Considering these factors, it is crucial to comprehensively evaluate the advantages and disadvantages of each method and optimize them for specific applications when selecting suitable REE extraction techniques. Future research should focus on developing more efficient, environmentally friendly, and cost-effective novel extraction processes [112]. Simultaneously, it is necessary to establish and improve recovery systems and regulations to promote the industrialization and scaling up of REE recycling, achieving a synergistic development of resource utilization and environmental protection [113].

## 4. Case Studies of Rare Earth Recovery from Typical E-Waste

The recovery of REEs from e-waste is crucial due to their critical role in modern technology and the environmental impacts of mining. This section explores case studies focusing on the recovery of REEs from waste permanent magnets and fluorescent powder waste, two of the most promising e-waste sources for REE recovery.

### 4.1. Waste Permanent Magnets: A Rich Source of REEs

Waste permanent magnets, particularly NdFeB magnets, are a significant source of REEs such as neodymium and dysprosium. The high concentration of these elements, along with the increasing demand for NdFeB magnets in applications like wind turbines and electric vehicles, makes them an attractive target for REE recovery [114,115,116]. In addition to their economic value, recovering REEs from waste permanent magnets also helps to mitigate the environmental impacts associated with primary mining and reduces the risk of supply disruptions [117,118].

### 4.2. Rare Earth Recovery from Waste Permanent Magnets

Fluorescent powders from spent lighting products contain high levels of valuable REEs such as yttrium, europium, and terbium. The proper handling and recycling of these materials are not only driven by their economic value but also by the need to comply with regulations regarding hazardous waste management [27,119]. Recovering REEs from fluorescent powder waste presents an opportunity to reduce the environmental footprint of the lighting industry and support the transition to a circular economy [16,120].

### 4.3. Comparison of REE Recovery Methods for Waste Permanent Magnets

Several methods have been investigated for recovering REEs from waste permanent magnets, each with its own advantages and challenges. Table 6 provides a comparison of the key aspects of these methods, including their efficiency, environmental impact, and scalability.

The case studies presented in this section demonstrate the potential for high recovery efficiencies and reduced environmental impact through the application of innovative methods such as supercritical fluid extraction, bioleaching, and near-zero-waste valorization. However, each method also faces specific challenges related to factors such as scalability, process optimization, and economic feasibility.

Traditional hydrometallurgical processes have been shown to achieve high purity and recovery rates, but their environmental impact remains a concern [121]. Bioleaching and near-zero-waste valorization offer more environmentally friendly approaches, but their efficiency and scalability need further improvement [118,122]. Supercritical fluid extraction has shown promising results in laboratory-scale studies, but its industrial applicability is yet to be demonstrated [123].

The choice of REE recovery method for waste permanent magnets ultimately depends on a balance between the desired efficiency, environmental sustainability, and economic viability. Further research and the optimization of these methods, as well as the development of novel hybrid processes, will be crucial for the sustainable recovery of REEs from waste permanent magnets. The challenges and future perspectives associated with these methods will be discussed in more detail in the following sections.

### 4.4. Advancements in REE Recovery from Fluorescent Powder Waste

Recent studies have explored various approaches for extracting REEs from waste fluorescent powders, aiming to improve recovery rates, selectivity, and environmental sustainability.

#### 4.4.1. Enhanced Leaching and Extraction Processes

Optimizing leaching parameters, such as acid concentration and temperature, has been shown to significantly enhance REE recovery rates from fluorescent powders [124,125]. For instance, a study utilized machine learning to guide the leaching process, focusing on parameters like particle size, which resulted in improved efficiency in extracting REEs from waste fluorescent lamps [27]. Ionic liquid extraction has emerged as a promising approach for selective REE recovery. Bifunctional ionic liquid extractants (Bif-ILEs) have shown high efficiencies and selectivities in extracting REEs. For example, a study demonstrated that using Bif-ILEs such as [A336][P204] and [A336][P507] achieved a recovery rate of 95.2% for REEs from a simulated waste fluorescent powder system [126]. This process involved a countercurrent extraction at specific phase ratios and pH levels, highlighting the potential of Bif-ILEs as efficient extractants for REE separation and recycling.

#### 4.4.2. Biohydrometallurgy: A Sustainable Approach

Biohydrometallurgy, which utilizes microbial activity to recover REEs, offers a more environmentally friendly alternative to traditional methods. This approach aligns with the goals of a circular economy by reducing the need for harsh chemicals and energy-intensive processes [127,128].

Microbial bioleaching is a key aspect of biohydrometallurgy, where microorganisms are employed to facilitate the leaching of REEs from waste materials. Autotrophic and heterotrophic microorganisms are used to generate acids that dissolve metals from industrial residues and electronic waste, including fluorescent lamp powders and spent catalysts. This method has been shown to be effective in reducing environmental impact compared to traditional chemical processes [127].

The recovery of REEs from industrial wastes using biohydrometallurgy has been explored extensively. Studies have demonstrated the potential of using specific microorganisms to bioleach REEs from materials such as e-waste and phosphor powders, which are significant sources of REEs like cerium, europium, and yttrium [127,129]. For example, a recent study investigated the bioleaching of REEs from fluorescent phosphor powder using the fungus Aspergillus niger CECT2807. This research demonstrated the effectiveness of using A. niger in both batch and semicontinuous systems to extract REEs from waste fluorescent lamp powders, highlighting the potential of fungal bioleaching as a sustainable and efficient method for recovering valuable metals from industrial waste streams [130].

Another study developed a scalable microbial platform for REE leaching and recovery from waste sources using the mesophilic methylotrophic bacterium Methylobacterium extorquens AM1. This research showed that M. extorquens AM1 could grow using electronic waste as a sole REE source, and the microbial platform could be scaled up to 10 L with consistent metal yields, demonstrating its potential for industrial applications [131].

Biohydrometallurgical processes are less energy-intensive and produce fewer pollutants than conventional methods, making them a viable option for sustainable mining and recycling practices. The application of biohydrometallurgy in REE recovery from waste materials contributes to the reduction in the ecological footprint associated with REE extraction.

However, further research is needed to optimize biohydrometallurgical processes for industrial-scale applications and to address challenges related to the efficiency and selectivity of REE recovery [132]. The development of advanced bioreactor systems and the identification of microbial strains with high leaching capabilities and specificity towards REEs are crucial areas for future research [131,132].

#### 4.4.3. Challenges and Opportunities in REE Recovery from Fluorescent Powder Waste

The recovery of REEs from fluorescent powder waste presents both challenges and opportunities. While advancements have been made in developing efficient and environmentally friendly extraction methods, there are still hurdles to overcome in terms of scalability and economic feasibility [19,133].

One of the main challenges is the heterogeneity of the waste material, which can affect the efficiency and selectivity of the recovery process. The development of advanced characterization techniques and pretreatment methods can help address this issue by providing a better understanding of the waste composition and enabling targeted extraction approaches [134].

Another challenge is the need for process integration and optimization to ensure the technical and economic viability of REE recovery from fluorescent powder waste. This requires a holistic approach that considers the entire value chain, from waste collection and sorting to the final purification and application of the recovered REEs [135].

Despite these challenges, the recovery of REEs from fluorescent powder waste offers significant opportunities for sustainable resource management and the development of a circular economy [134]. By reducing the reliance on primary mining and mitigating the environmental impact of waste disposal, REE recovery from secondary sources can contribute to a more sustainable and resilient supply chain [136].

To fully realize these opportunities, ongoing research and collaboration between academia, industry, and government are essential. This includes the development of novel extraction methods, the optimization of existing processes, and the implementation of supportive policies and regulations that promote the sustainable recovery and use of REEs.

## 5. Existing Problems and Future Perspectives

### 5.1. Urgent Need for Establishing and Improving Recovery Systems and Regulations

Despite the advancements in REE recovery technologies, there is still an urgent need to establish and improve recovery systems and regulations. Current recycling infrastructure and policies are insufficient to handle the increasing volume and complexity of e-waste containing REEs [137]. The lack of standardized collection, sorting, and processing procedures leads to inefficiencies and limits the potential for large-scale REE recovery [138].

To address these issues, governments and industries must collaborate to develop comprehensive e-waste management strategies that prioritize REE recovery. This includes implementing extended producer responsibility (EPR) schemes, which hold manufacturers accountable for the end-of-life management of their products [139]. Additionally, creating incentives for consumers to properly dispose of e-waste and investing in public awareness campaigns can help improve collection rates and reduce the amount of REEs lost to landfills [140].

**Figure 9:**
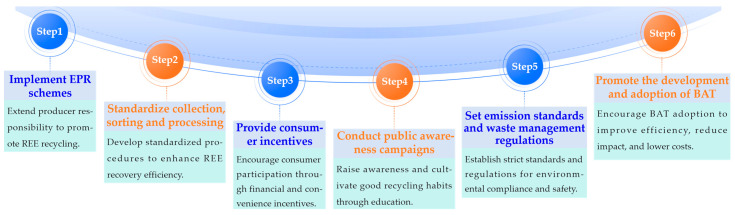
Key steps in establishing and improving REE recycling systems and regulations.

Figure 9 illustrates the key steps in establishing and improving REE recycling systems and regulations. As shown in Figure 8, establishing a comprehensive REE recycling system and regulations requires multi-faceted efforts, including implementing EPR schemes, standardizing collection, sorting, and processing procedures, providing consumer incentives, conducting public awareness campaigns, setting emission and waste management standards, and promoting best available technology (BAT) adoption.

Establishing clear regulations and guidelines for REE recovery processes is also crucial to ensure environmentally sound practices and protect public health [141]. This involves setting standards for emissions, waste management, and occupational safety in the recycling industry [142]. Furthermore, promoting the development and adoption of BAT can help minimize the environmental impact of REE recovery while maximizing resource efficiency [143].

The integrated implementation of these measures will contribute to enhancing the efficiency of REE recovery, reducing environmental impacts, and ultimately promoting the sustainable utilization of REEs from e-waste. Building upon these efforts to establish and improve recovery systems and regulations, the following section will discuss the optimization and integration of recovery technologies to further advance the sustainable development of the rare earth recycling industry.

### 5.2. Optimization and Integration of Recovery Technologies

While Section 4 discussed various REE recovery technologies and their optimization, there is still a need for further integration and synergy between these methods. Each recovery technology has its strengths and limitations, and combining them in innovative ways can lead to more efficient, cost-effective, and environmentally friendly processes [144].

**Figure 10:**
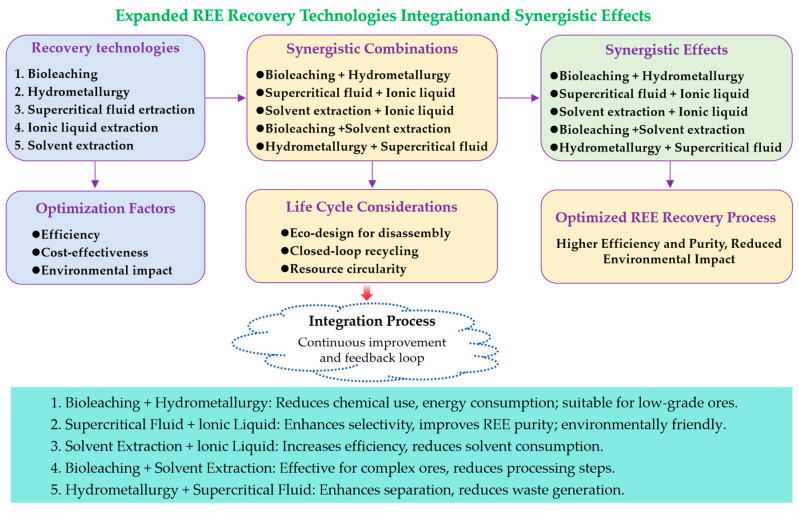
Schematic diagram of REE recovery technology optimization and integration.

The image below (Figure 10) presents an overview of the expanded REE recovery technologies, their synergistic combinations, and the resulting synergistic effects. It also highlights the optimization factors, life cycle considerations, and the continuous improvement and feedback loop in the integration process.

As shown in Figure 10, several synergistic combinations can enhance the overall performance of REE recovery processes. For example, integrating bioleaching with hydrometallurgy can reduce chemical usage, energy consumption, and environmental impact while maintaining high recovery rates [127]. Similarly, combining supercritical fluid extraction with ionic liquid extraction may enhance the selectivity and purity of recovered REEs [145]. Other promising combinations include solvent extraction + ionic liquid extraction, bioleaching + solvent extraction, and hydrometallurgy + supercritical fluid.

The optimization of these integrated recovery technologies should consider factors such as efficiency, cost-effectiveness, and environmental impact. Life cycle considerations, including eco-design for disassembly, closed-loop recycling, and resource circularity, are also crucial in developing sustainable REE recovery processes [146,147].

Moreover, the integration process should involve continuous improvement and a feedback loop to refine and adapt the combined technologies based on their performance and evolving market demands. This iterative approach ensures that the integrated recovery processes remain optimized and responsive to changing conditions.

Researchers and industries should explore these synergistic approaches to develop hybrid recovery processes that capitalize on the advantages of different technologies. By doing so, they can contribute to the development of more sustainable and efficient REE recovery methods, ultimately supporting the growing demand for these critical materials in various applications.

To fully realize the potential of these optimized and integrated recovery technologies, it is essential to consider their environmental impacts and strive for a balance between resource utilization and environmental protection, which will be discussed in the following section.

### 5.3. Synergetic Development of Resource Utilization and Environmental Friendliness

Achieving sustainable development in the rare earth recycling industry necessitates striking a delicate balance between resource utilization and environmental protection [148]. While the recovery of REEs from e-waste offers significant economic opportunities, it must not compromise ecological integrity or public health [149].

**Figure 11:**
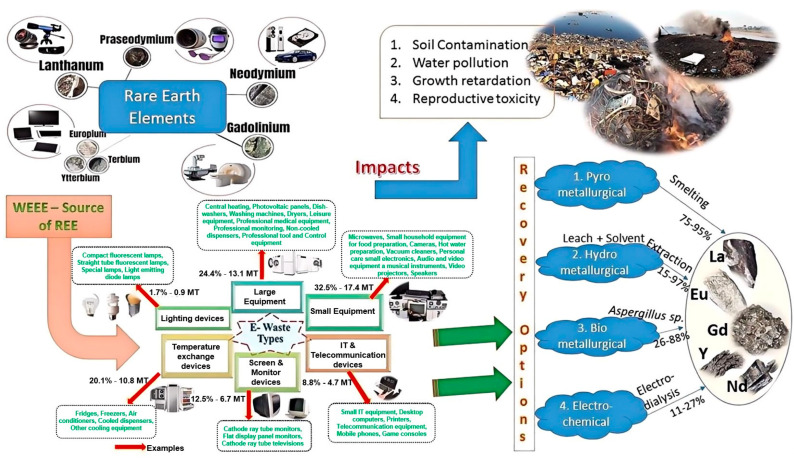
Sources, distribution, and environmental impacts of REEs in e-waste and their recycling process [150].

Figure 11 illustrates the sources and distribution of rare earth elements in electronic waste, as well as the potential environmental impacts of the recycling process [150]. As shown in the figure, the recycling of REEs from e-waste can lead to various environmental issues, such as soil and water pollution, growth inhibition, and reproductive toxicity. These environmental impacts highlight the importance of adopting sustainable practices and technologies in the rare earth recycling industry.

The “Recovery Options” section on the right side of Figure 11 illustrates various methods for recovering rare earth elements (REEs), including pyrometallurgical, hydrometallurgical, bio-metallurgical, and electro-chemical methods. Each method has its own advantages and disadvantages, necessitating a comprehensive evaluation of factors such as recovery efficiency, environmental impact, and economic feasibility [82,151].

By carefully selecting and optimizing these recovery methods, the environmental impacts of the recycling process can be minimized while maximizing resource utilization efficiency. The adoption of circular economy principles, which emphasize the maximization of resource use efficiency and the minimization of waste generation, is crucial in this context. This entails the design of easily recyclable products [152], the development of effective collection and sorting infrastructure [153], and the promotion of recycled REE utilization in new applications [154]. The successful implementation of such strategies, as exemplified by the EU’s WEEE Directive, can drive the sustainable growth of the REE recycling industry [152,153].

The continuous monitoring and mitigation of the environmental impacts associated with REE recycling processes are also imperative. This involves the implementation of robust emission control measures, the proper management of hazardous waste, and investment in green technologies that minimize energy and water consumption. The application of LCA tools can aid in identifying environmental hotspots and guiding process optimization efforts [155,156].

Governments play a pivotal role in promoting the sustainable development of the REE recycling industry. This can be achieved through policy support, financial incentives, and the establishment of conducive regulatory frameworks. Collaborative efforts between businesses, governments, and the public are essential to foster a circular economy approach and prioritize environmental stewardship [157].

By synergistically advancing resource utilization efficiency and environmental sustainability, the rare earth recycling industry can contribute significantly to the creation of a more resilient and circular economy.

## 6. Conclusions

This comprehensive review has shed light on the immense potential of e-waste as a secondary source of rare earth elements, with a particular focus on waste permanent magnets and fluorescent powders. The in-depth analysis of the factors influencing REE distribution in e-waste, coupled with the systematic evaluation of various extraction techniques, provides a solid foundation for the development of effective and sustainable recovery strategies. The case studies presented demonstrate the feasibility and promise of different REE recovery methods, ranging from traditional hydrometallurgical processes to emerging green technologies such as bioleaching and supercritical fluid extraction.

However, the path to fully realizing the potential of REE recovery from e-waste is fraught with challenges, including technical barriers, economic viability concerns, and environmental sustainability considerations. Overcoming these challenges requires a multifaceted approach that encompasses the establishment of robust recovery systems and regulations, the optimization and integration of extraction technologies, and the synergetic development of resource utilization and environmental protection. Future research should focus on developing eco-friendly and efficient recovery processes while also considering the entire life cycle of REEs to minimize environmental burdens. Specifically, we recommend investigating novel extraction technologies that balance efficiency with sustainability, optimizing the integration of various recovery methods for more holistic approaches, and conducting comprehensive life cycle assessments of REE recovery processes.

The success of this endeavor hinges on the collaborative efforts of all stakeholders, including researchers, industry practitioners, policymakers, and consumers. By fostering innovation, promoting sustainable practices, and encouraging responsible consumption, we can collectively work towards a more circular and resilient economy that values the sustainable utilization of critical resources like REEs. As we navigate the complexities of REE recovery from e-waste, it is crucial to prioritize the development of cost-effective, environmentally sound, and socially responsible extraction methods. Additionally, exploring the potential of emerging fields such as machine learning and artificial intelligence in optimizing REE recovery processes could yield significant advancements.

In conclusion, this review underscores the significance of recovering REEs from e-waste and highlights the current state of knowledge in this field. It serves as a clarion call for further research, innovation, and collaboration to advance the sustainable recovery of these critical elements. Through concerted research efforts, supportive policies, and multi-stakeholder engagement, we can unlock the full potential of this valuable secondary resource and pave the way for a more sustainable future. By embracing the challenges and opportunities presented by e-waste recycling, we can move closer to a circular economy that optimizes resource use, minimizes environmental impacts, and ensures the long-term availability of REEs for future generations.

## Figures and Tables

**Table 1:** Typical REE content in various types of e-waste.

E-Waste Type	Main Components	REEs Present	Concentration (ppm)	Ref.
Hard disk drives	Permanent magnets	Nd, Dy, Pr	2500–15,000	[13,22]
Fluorescent lamps	Phosphors	Y, Eu, Ce, Tb, La	1000–20,000	[22,23]
Smartphones	Screens, speakers, vibration motors	La, Ce, Pr, Nd, Sm, Eu, Gd, Tb, Dy, Y	10–1000	[23]
Computers	Printed circuit boards, hard disk drives, speakers	La, Ce, Pr, Nd, Sm, Eu, Gd, Tb, Dy, Y	10–1000	[24]
Televisions	Screens, speakers	Y, Eu, Tb	10–100	[13,24]

**Table 2:** Overview of physical separation techniques for REE recovery from E-waste.

Technique	Principle	Advantages	Applications	Efficiency/Considerations	Ref.
Manual Disassembly	Hand separation of REE-containing components	High precision, suitable for high-value components	Recovery of NdFeB magnets from hard disk drives	Labor-intensive but effective for valuable parts	[47]
Magnetic Separation	Separation based on magnetic properties	Efficient separation of magnetic materials	Isolation of NdFeB magnets from other e-waste materials	Most effective for REE-containing magnetic materials	[48]
Density-Based Separation	Separation based on density differences	Can separate components with different densities	Gravity separation or flotation	Suitable for materials with significant density differences	[49]
Size Reduction and Sieving	Particle size reduction and classification by size	Improves efficiency of subsequent processing	Liberation and classification of REE-containing materials	Prepares materials for chemical processing, enhancing efficiency	[50]

**Table 3:** Overview of hydrometallurgical techniques for REE extraction from e-waste.

Technique	Principle	Advantages	Applications	Ref.
Acid Leaching	Dissolution of REEs using acidic solutions	High leaching efficiency, selectivity	Extraction of REEs from NdFeB magnets, fluorescent phosphors	[58,59]
Solvent Extraction	Selective separation of REEs using organic extractants	High purity, efficient separation of individual REEs	Purification of leach solutions containing multiple REEs	[59,60]
Precipitation	Formation of solid REE compounds by pH adjustment or addition of precipitating agents	Simple, cost-effective	Recovery of REEs as oxalates, carbonates, or hydroxides	[61,62]

**Table 4:** Overview of pyrometallurgical techniques for REE recovery from e-waste.

Technique	Principle	Advantages	Applications	Ref.
Molten salt electrolysis	Electrochemical extraction of REEs using high-temperature molten salts	Efficient separation of REEs from complex matrices	Recovery of REEs as oxalates, carbonates, or hydroxides	[78]
Roasting	High-temperature conversion of REE compounds to oxides	Improves leaching efficiency, enables selective recovery	Treatment of REE phosphors and oxides	[79]
Smelting	Melting e-waste at high temperatures to concentrate REEs in molten phase	Enables recovery from metallic components, high throughput	Purification of leach solutions containing multiple REEs	[75,81]

**Table 5:** Comparison of REE recovery methods from e-waste.

Method	Recovery/%	Recovered Form	Env. Impact	Energy Use	Cost-Effectiveness	Pros/Cons	Ref.
Traditional hydrometallurgy	80–99%	Metal salts, pure metals	Moderate chemical waste	Moderate	High	+High efficiency −Chemical-intensive	[104]
Bioleaching	40–90% *	Metal ions in solution	Low	Low	Moderate	+Eco-friendly −Time-consuming	[105]
Supercritical fluid extraction	80–95%	Metal complexes	Low waste generation	High	Moderate	+High purity −High pressure required	[106]
Ionic liquid extraction	85–98%	Metal ions in ionic liquid	Low volatility, reusable	Low	High initial cost	+Selective extraction −High cost of ionic liquids	[107]
Pyrometallurgy	80–95%	Metal alloys, pure metals	High energy use, emissions	Very high	Moderate	+High throughput −High energy use	[27]

* Efficiency varies widely depending on specific REEs, source material, and leaching conditions.

**Table 6:** Comparison of REE recovery methods from waste permanent magnets.

Method	Efficiency	Environmental Impact	Scalability	Example	Ref.
Traditional Hydrometallurgical	High	High	Established	MSX process (99.5% purity, >95% recovery)	[121]
Bioleaching	Moderate	Low	Developing	Aspergillus niger bioleaching (1.37 mg/L REEs)	[122]
Supercritical Fluid Extraction	High	Low	Laboratory scale	SCFE with organophosphorus reagents (TEP, TBP, TBPO, TOPO)	[123]
Near-Zero-Waste Valorization	High	Low	Developing	Valorization with antimicrobial beads (87.21% inhibition)	[118]

## Abbreviations

REE(s)	Rare earth element(s)
E-waste	Electronic waste
D2EHPA	Di-(2-ethylhexyl) phosphoric acid
PC-88A	2-ethylhexyl phosphonic acid mono-2-ethylhexyl ester
TBP	Tributyl phosphate
SFE	Supercritical fluid extraction
scCO_2_	Supercritical carbon dioxide
ILs	Ionic liquids
[A336][P204]	Bifunctional ionic liquid extractant
[A336][P507]	Bifunctional ionic liquid extractant
[C101][SCN]	Trihexyl(tetradecyl)phosphonium thiocyanate
[A336][SCN]	Tricaprylmethyl ammonium thiocyanate
TEP	Triethyl phosphate
TBP	Tributyl phosphate
TBPO	Tributyl phosphine oxide
TOPO	Tri octyl phosphine oxide
CD	Circular dichroism
NMR	Nuclear magnetic resonance
MD	Molecular dynamics
ITC	Isothermal titration calorimetry
QCM-D	Quartz crystal microbalance with dissipation
QCM-D	Quartz crystal microbalance with dissipation
LCA	Life cycle assessment
BAT	Best available technologies
EPR	Extended producer responsibility
WEEE	Waste electrical and electronic equipment
